# MINORITY AGING BEYOND THE CLASSROOM

**DOI:** 10.1093/geroni/igz038.1314

**Published:** 2019-11-08

**Authors:** Tiffany R Washington

**Affiliations:** University of Georgia, Athens, Georgia, United States

## Abstract

Service-learning is the pedagogical approach of integrating classroom learning objectives with community-based experiences. In gerontology education, service-learning is one way educators can collaborate with their respective communities to expose students to topics in minority aging. This paper presentation describes students’ experiences in a service-learning gerontological social work course embedded in a university-community partnership that took students beyond the classroom and into the community to learn about minority aging. In-classroom topics included social injustices in aging, caregiving in African American families, culturally-tailored caregiving interventions, and health disparities. In the community, students conducted in-home visits engaging persons with dementia in a tailored activities, thus freeing caregivers to engage in self-care activities. Classroom and community experiences were connected through ongoing reflection, critical thinking, and problem solving activities. Survey data revealed students experienced increased gerontological self-efficacy and increased knowledge and attitudes about dementia. This study has implications for future course design around minority aging.

